# Dickkopf1 Regulates Fate Decision and Drives Breast Cancer Stem Cells to Differentiation: An Experimentally Supported Mathematical Model

**DOI:** 10.1371/journal.pone.0024225

**Published:** 2011-09-06

**Authors:** Zvia Agur, Oleg U. Kirnasovsky, Genadiy Vasserman, Lilach Tencer-Hershkowicz, Yuri Kogan, Hannah Harrison, Rebecca Lamb, Robert B. Clarke

**Affiliations:** 1 Institute for Medical BioMathematics, Bene Ataroth, Israel; 2 Breast Biology Group, School of Cancer and Enabling Sciences, Paterson Institute for Cancer Research, University of Manchester, Manchester, United Kingdom; Northwestern University Feinberg School of Medicine, United States of America

## Abstract

**Background:**

Modulation of cellular signaling pathways can change the replication/differentiation balance in cancer stem cells (CSCs), thus affecting tumor growth and recurrence. Analysis of a simple, experimentally verified, mathematical model suggests that this balance is maintained by quorum sensing (QS).

**Methodology/Principal Findings:**

To explore the mechanism by which putative QS cellular signals in mammary stem cells (SCs) may regulate SC fate decisions, we developed a multi-scale mathematical model, integrating extra-cellular and intra-cellular signal transduction within the mammary tissue dynamics. Preliminary model analysis of the single cell dynamics indicated that Dickkopf1 (Dkk1), a protein known to negatively regulate the Wnt pathway, can serve as anti-proliferation and pro-maturation signal to the cell. Simulations of the multi-scale tissue model suggested that Dkk1 may be a QS factor, regulating SC density on the level of the whole tissue: relatively low levels of exogenously applied Dkk1 have little effect on SC numbers, whereas high levels drive SCs into differentiation. To verify these model predictions, we treated the MCF-7 cell line and primary breast cancer (BC) cells from 3 patient samples with different concentrations and dosing regimens of Dkk1, and evaluated subsequent formation of mammospheres (MS) and the mammary SC marker CD44^+^CD24^lo^. As predicted by the model, low concentrations of Dkk1 had no effect on primary BC cells, or even increased MS formation among MCF-7 cells, whereas high Dkk1 concentrations decreased MS formation among both primary BC cells and MCF-7 cells.

**Conclusions/Significance:**

Our study suggests that Dkk1 treatment may be more robust than other methods for eliminating CSCs, as it challenges a general cellular homeostasis mechanism, namely, fate decision by QS. The study also suggests that low dose Dkk1 administration may be counterproductive; we showed experimentally that in some cases it can stimulate CSC proliferation, although this needs validating *in vivo.*

## Introduction

There is growing evidence that cancer arises from self–renewing malignant SCs – known also as “cancer initiating cells” or “stem-like cells” – that are resistant to standard therapy [1]. Such cells have been amply documented in BC and are considered to be a reason for the failure of therapy and a source of relapse [2], [3].

Understanding the key molecular mechanism governing the transition of BC-SCs from self-renewal to differentiation could indicate how to manipulate fate decision in these cells, so as to drive most or all of the proliferating cells into differentiation. The resulting differentiated cells would have a significantly reduced replication capacity and would be more sensitive to standard therapy and thus easier to eliminate [4].

BC-SCs' self-renewal is mediated by different signaling pathways, mainly by Wnt [5], [6], Notch [7], and E-cadherin [8] (see detailed description in the Materials and Methods and reviews in Refs. [9], [10], and [11]). These signaling pathways are complex, involving an intricate interplay of negative and positive stimuli, which has complicated researchers' attempts to comprehend the system as a whole. Traditional experimental biology paradigms do not suffice here, thus calling for new methodologies for disentangling SC control mechanisms.

Previously, we constructed and analyzed a general mathematical model for developing tissues. Based on model analysis we asserted that the balance between replication and differentiation is mainly controlled by a negative feedback on SC proliferation (denoted QS*;* [12], [13]). This mathematical result was supported by laboratory experiments showing that, as the QS theory suggests and independently of the initial SC fraction, there is a fixed proportion of SCs (marked by either CD44 or CD44+/24lo/ESA+) at population confluence [14].

In this work we investigated QS in CSCs by developing a new relatively simple mathematical model for the major intra-cellular processes regulating fate decision in mammary SCs, and implementing it within the general developing tissue model. Using this approach we explored the interactions between events occurring on the intra-cellular scale and events occurring on the tissue scale, and examined how the cellular pathways might serve as a control system that integrates signals from the environment to regulate cell fate. Preliminary analysis of the cell model has singled out Dkk1 as a plausible mechanism, whose modulation can divert a BC-SC from proliferation into differentiation [12]. However, it was not a priori evident that the same mechanism would be effective when the dynamics at the tissue scale was to be examined, since multiple, non synchronous feedbacks could drive the larger system to an unexpected outcome. Therefore, we simulated the combined multi-scale mathematical model to study the role of Dkk1 in BC-SC regulation, in order to predict the conditions, within the tumor environment, which divert BC-SCs from proliferation.

Our mathematical model, fully presented in this work (below and in Text, S1), provides a general theory for the role of Dkk1 in SC regulation, and predicts that Dkk1 in the inter-cellular space can balance proliferation and differentiation of SCs through its effect on the Wnt and, indirectly, on the Notch pathways. Our model further predicts that the addition of small doses of exogenous Dkk1 will not decrease, and may even slightly increase, mammary cell proliferation, whereas large doses of Dkk1 will divert proliferating SCs to differentiation. We present herein experimental results in MCF-7 cells and in BC cells from a patient's biopsy, which support the predictions of the mathematical model.

## Results

### Mathematical model

Our model is a multi-scale tumor model, aimed at exploring the interactions between events occurring on the molecular scale and events occurring on the tissue scale, and at examining how the cellular pathways can serve as a system that integrates signals from the environment to regulate cell fate.

### Modelling the tissue dynamics

In order to model the structure of the tissue, containing both continuous protein activities and discrete cellular developmental and spatial states, we used a *hybrid cellular automata* (HCA) formalism,[15] and added to it a random choice of the daughter cell settling sites [13], [16]. Our tissue model is a 2-dimensional HCA grid of 800 cells. Each SC on the grid has the form of our SC model described below.

The full description of our cellular automata model can be found in [12], [13], [16] In brief, our tissue model assumes that SCs, whose cell cycle duration is τ, can divide symmetrically, creating new SCs, and alternatively, can differentiate. The third possibility, namely, division of a SC to two differentiated cells is ignored for simplicity of the model. SC decision is determined by the SC's intra-cellular protein levels that are influenced by micro-environmental interactions, as detailed below. When no vacant neighboring sites are available for replicating into, a SC will remain quiescent. The differentiated cell compartment includes all differentiated descendants of a SC, whose life span, Φ, is limited and whose brief proliferation is ignored here, for simplicity. A cell that dies creates an empty grid site, which can become occupied by a proliferating adjacent cell.

### Modelling signal transduction

**Figure 1:**
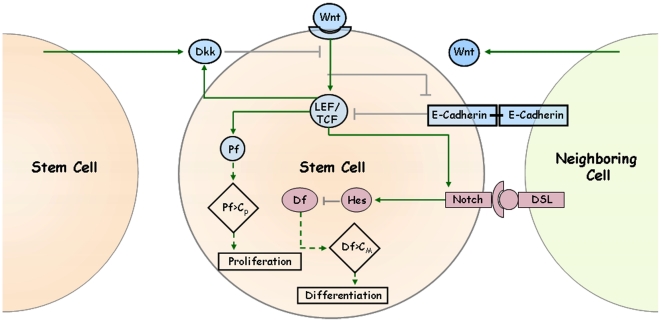
Schematic representation of the mathematical model of fate decision by breast cancer stem cells. Wnt signaling pathway induces LEF/TCF activation, resulting in up-regulation of Pf, Dkk1 and Notch signaling activators. Wnt signaling pathway also down-regulates E-cadherins. Dkk1 and E-cadherins inhibit Wnt signaling pathway. Activation of Notch signaling pathway by DSL up-regulates Hes expression, which inhibits Df. Pf, proliferation factor; Df, Differentiation factors; C_P_, Pf threshold; C_M_, Df threshold; DSL, Delta, Serrate, Lag-2; Dkk1, Dickkopf1 protein. Pale blue – Wnt protein; purple – Notch pathway protein; green arrow – activation; grey arrow – inhibition; dashed arrow – threshold-dependent effect.

We formulated the mathematical model of mammary SCs fate decision, based on literature information (see “Biological background for the mathematical model” in SI), suggesting that Wnt signaling activates LEF/TCF protein complex leading to up-regulation of proliferation factors (PF), Dkk1 and proteins involved in the Notch signaling pathway. The Wnt pathway can down-regulate E-cadherins, whereas Dkk1 and E-cadherins can inhibit Wnt signaling. Activation of Notch receptor by a transmembrane ligand DSL, presented by an adjacent cell, causes up-regulation of Hes, which inhibits the differentiation factors (DF). Notch activity can be further stimulated by LEF/TCF (See Fig. 1 for graphical representation).

The intracellular model is formulated as a set of coupled ordinary differential equations, describing the dynamics of the proteins comprising the modeled regulatory pathways. The main model equations and their short explanation are given below.



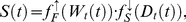
(1)




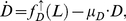
(2)




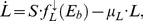
(3)




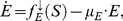
(4.1)




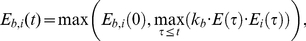
(4.2)




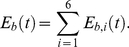
(4.3)




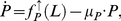
(5)




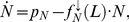
(6.1)




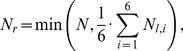
(6.2)




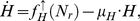
(7)




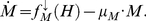
(8)


In all the equations, 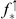 denote increasing functions; 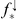 - decreasing functions. In Eq. 1, 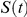 denotes signal intensity of the Wnt pathway in a cell at time *t*; 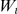 and 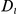 are total levels of Wnt and Dkk1 proteins, respectively, in the close environment of the cell; 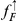 – Wnt signal intensity as a function of 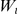; 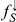 – inhibition of the Wnt signal by Dkk1, as a function of 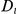. In Eq. 2, 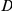 denotes the level of Dkk1 produced by the cell; 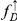 – dependence of Dkk1 synthesis rate on activated LEF/TCF; 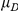 – Dkk1 degradation rate. In Eq. 3, 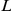 denotes the level of activated LEF/TCF; 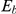
– E-cadherin bound to neighboring cells; 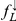 – dependence of LEF/TCF activation rate on the bound E-cadherin;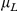 - LEF/TCF degradation rate. In Eq. 4.1, 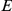 denotes the total E-cadherin level; 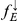 – dependence of E-cadherin synthesis rate on 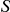;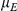 – E-cadherin degradation rate. In Eq. 4.2, 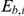 denotes the level of the E-cadherin bound to the neighbouring cell into 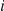-th direction; 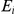 – level of E-cadherin in the neighbouring cell situated in 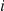-th direction from the considered cell;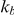 – E-cadherin binding coefficient. In Eq. 4.3, the total level of bound E-cadherins in the cell is computed as a sum of E-cadherins bound to all the six neighbouring cells. In Eq. 5, 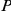 denotes the level of PF; 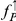 – dependence of PF synthesis rate on 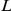;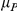 – PF degradation rate. In Eq. 6.1, 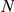 denotes Notch receptor level; 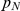
– Notch receptor synthesis rate; 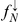 – dependence of Notch receptor degradation rate on 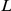. In Eq. 6.2 the level of activated Notch receptors, 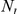, is computed as a minimum between the total Notch receptor level and the level of DSL receptors available from all the adjacent cells, denoted by 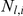 for the adjacent cell in *i*-th direction. In Eq. 7, 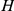 denotes HES level; 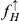
– dependence of HES synthesis rate on 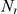; 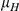 – HES degradation rate. In Eq. 8, 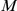
denotes level of DF; 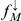 – dependence of DF synthesis rate on 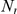;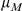 – DF degradation rate. For the complete mathematical model and the detailed explanation of every equation, see SI.

It is important to bear in mind that the effect of Notch and Wnt on the proliferation/differentiation balance is taken account of by the balance between the amount of PF and DF regulated by the former and latter biochemical pathways, respectively. The synthesis of PF is up-regulated by the activated LEF/TCF transcription factors. Degradation of PF and DF is proportional to their level (first order degradation). The cell replicates upon PF accumulation above a certain threshold,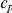 and the availability of an adjacent empty site. The switch to differentiation is induced when DFs reach a certain threshold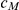. Note that in our model, SCs transit to quiescence when the levels of the proliferating factors are below the threshold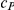, but differentiating factor levels have not reached 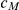.

### Mathematical model simulations - effects of Dkk1 on SC proliferation

Our primary interest in this paper was to examine SC response to changes in the local environment, such as increase or decrease in the number of neighbors within the tissue, or addition of regulatory molecules to the environment. In terms of our suggested model, such events will produce feedbacks, which are realized in some of the signals received by the SC. These signals include the level of external Dkk1 and the level of external Wnt entering the cell, the number of DSL receptors on the surface of neighboring cells, as well as the number of E-cadherins on neighboring cells, which determines the proportion of bound E-cadherins. Our preliminary analysis of a single SC behavior suggests that an increase in the concentration of either Dkk1 or E-cadherin decelerates proliferation as well as accelerating differentiation. From [12], it also follows that reducing Wnt or DSL could yield a similar outcome. For practical reasons, here we focused attention on the addition of Dkk1, as the number of receptors on the cell surface cannot be externally controlled and the other ligands are also less controllable. However, other anti-Wnt pathway treatments are currently investigated (Kogan et al., in preparation).

**Figure 2:**
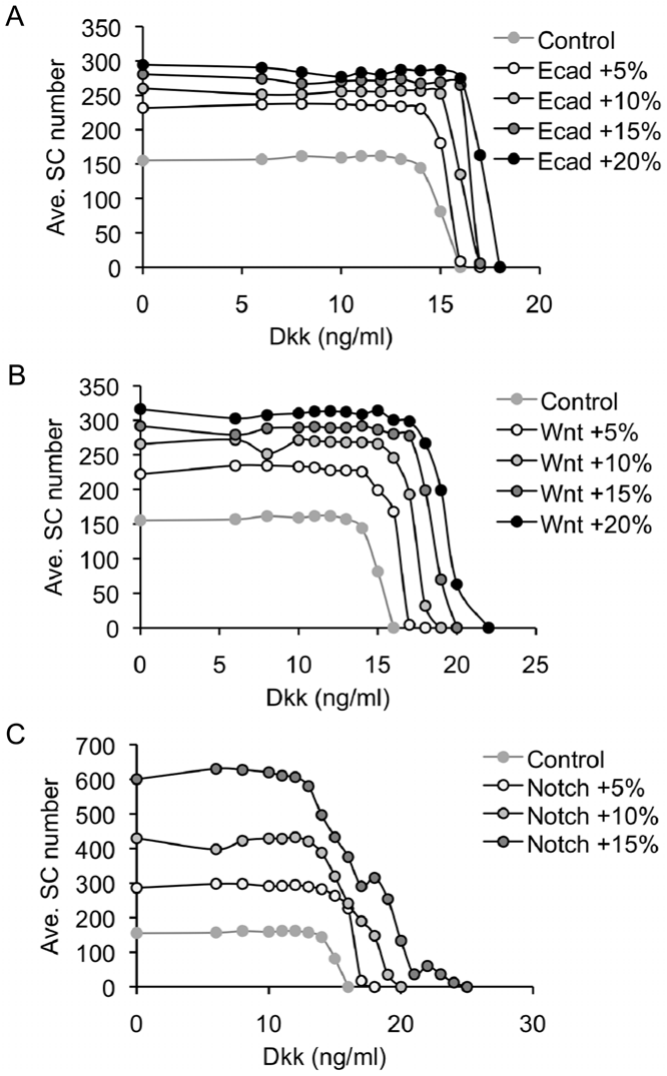
Simulations of Dkk1 effects on number of SCs in normal and mutated mammary tissue. Continuous administration of Dkk1, 0 to 25 ng/mL, for 48 h to normal or mutated mammary tissue was simulated. The model was simulated over three months after Dkk1 treatment. The average number of SCs, over the last three simulated days, was calculated over 50 simulation experiments for each combination of concentration and cell type. **A.** Cells bearing mutations in the amount of E-cadherins required for inhibiting LEF/TCF activation, vs. normal cells. **B.** Cells bearing mutations increasing Wnt ligand expression vs. normal cells. **C.** Cells bearing mutations increasing Notch receptor synthesis vs. normal cells.

We simulated the effects of Dkk1 on cell proliferation in the normal mammary tissue. The results indicate that the number of SCs in culture is independent, to any significant extent, of the concentrations of Dkk1 that are lower than 13 ng/ml. Above this concentration, the number of SCs, both proliferating and quiescent, rapidly decreased, due to differentiation (grey full circles in Fig. 2). That in our simulations SCs are eliminated only by transition to differentiation, is self-evident from the structure of the model, not allowing for SC apoptosis.

Our simulations further suggest that the dose effect of Dkk1 on SC proliferation depends on the particular signaling defects. Association of defective Wnt and Notch signaling with BC points to their possible role in carcinogenesis [17]. We simulated Dkk1–dependent proliferation in BC-SCs characterized by aberrant activity in the signal transduction pathways. For that, we “tittered in” the effects of the respective molecular defects in the modeled signaling pathways, on Wnt/Noch activation or on E-cadherin loss, and observed the effects. Mutations in Notch and Wnt signaling pathways were simulated by 5 to 20 percent increase in the rate of Notch receptor synthesis and the Wnt ligand expression level, respectively. Mutations in E-cadherin were simulated by 5 to 20 percent increase in E-cadherin concentration, required for inhibition of LEF/TCF activity (see explanation in the Materials and Methods section). Our simulation results show that increasing the level of these molecules, which represent mutation-driven activation of signaling pathways, did not change the qualitative dependence on Dkk1, but did increase the number of BC-SCs in culture (Fig 2). As in normal cells, here too the effect of Dkk1 was biphasic. Below a threshold of Dkk1 concentration, there was no significant Dkk1 effect. Above that threshold concentration, which was somewhat higher than for normal cells, BC-SC number decreased in a Dkk1 dose dependent manner (Fig. 2). It can be seen in Fig. 2 that this threshold Dkk1 concentration increases with the expression level of the signaling proteins induced by the particular mutations. In addition, under relatively low Dkk1 levels, the rate of SC proliferation increased with increasing aberration in the rate of Notch receptor synthesis.

Taken together, our model simulations show that, whereas addition of small amounts of Dkk1 do not change or sometimes even increase SC numbers, there exists a threshold of exogenous Dkk1 concentration, above which SC numbers are reduced, through SC differentiation in a dose-dependent and a mutation-dependent manner, until all SCs undergo differentiation.

### Experimental testing of model predictions

**Figure 3:**
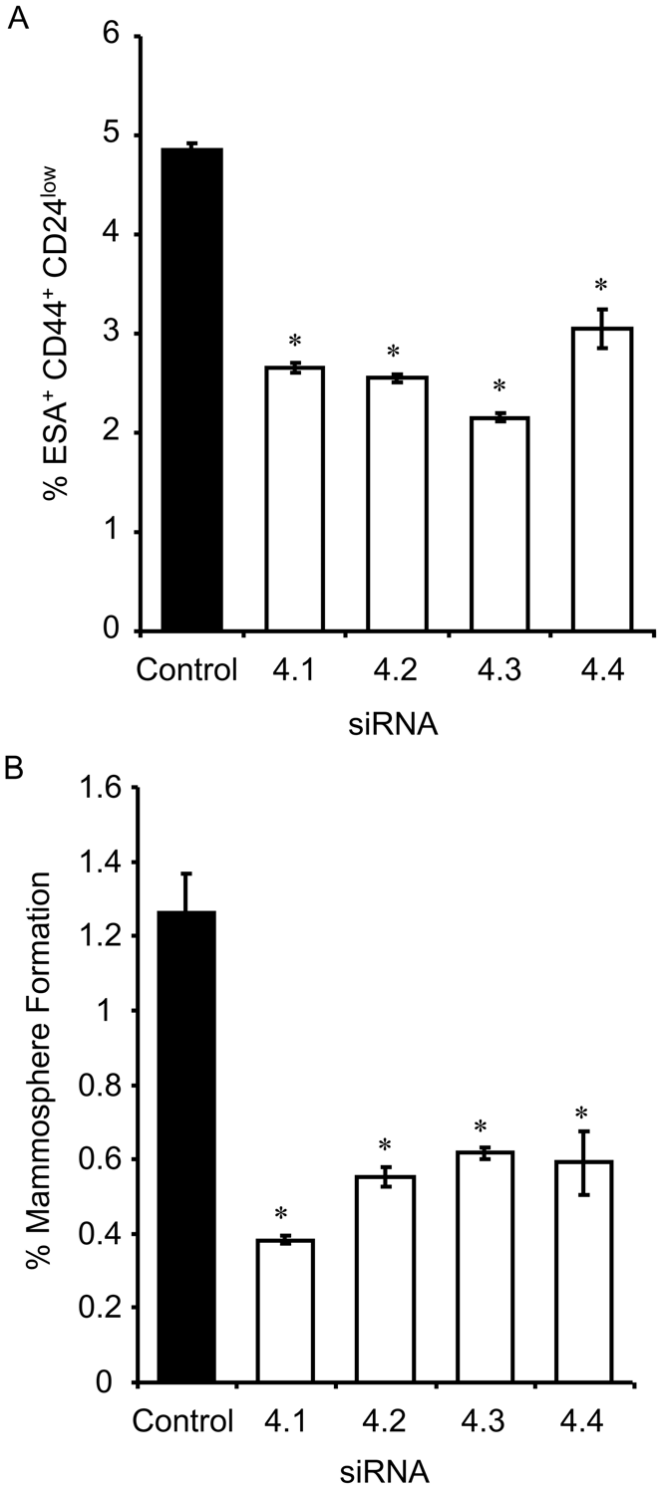
Effects of Notch4 on percentage CD44^+^ CD24^low^ cells and mammosphere formation. siRNA designed to four unique sites in the Notch4 receptor were used to inhibit Notch signalling. A non-specific siRNA was used as a control for transfection. siRNA, was trasfected to MCF7 cells in monolayer for 72 h before analysis of CD44^+^CD24^-/low^ cell surface markers (A), or mammosphere forming assay (B). Data collected from three independent experiments in A or two independent experiments in B are represented as mean ± SEM. Asterisks mark statistically significant differences (*p*≤0.05 by I-test).

The mathematical model simulations suggested that increasing Notch receptor activity will increase BC-SCs numbers (Fig. 2C). We used the long established ER+ breast cancer cell line MCF-7 and measured the effect of recombinant human Notch-receptor ligand DLL4 (rhDLL4) on the proportion of BC cells characterized by stem-cell phenotype CD44+CD24-/low[1], [7], and on MS formation, as a measure of SC number; [7], [18] MS are colonies formed in suspension from single cells and therefore measure clonogenic potential; cf. ref. [7]). We found that increasing the Notch receptor activity by addition of rhDLL4 increased the MCF-7 cell line MS formation and numbers of CD44+CD24-/low cells (Table 1; P<0.05). In keeping with this positive effect of Notch activity on BC-SCs number, we tested the effect of DAPT, a γ-secretase inhibitor that blocks Notch receptor activity. DAPT reduced both MS formation and the percentage of CD44+CD24-/low cells (Table 2; P<0.05). High activity of Notch4 in BC-SCs was recently detected in CD44+CD24low cells by the presence of relatively high levels of the cleaved Notch4 intra-cellular domain [19]. To confirm the role of Notch activation in BC-SCs, we knocked down Notch4 expression by siRNA. Following treatment with all siRNA sequences, the CD44+CD24-/low population (Fig. 3A) and number of MSs formed (Fig. 3B) were significantly reduced.

**Table 1:** Effects of the Notch receptor ligand rhDLL4 on CD44^+^ CD24^low^ expression and mammosphere formation.

rhDLL4 Exposure (Hours)	Fold Change Mammosphere formation	Fold Change CD44+ CD24low
24	2.23±0.01*	1.59±0.03*
48	2.4±0.22*	1.49±1.11*

* p<0.05, data collected from 3 independent experiments and represented as mean ± SEM.

Recombinant human Delta4 (rhDLL4) in gelatin was adsorbed to tissue culture plates before plating of MCF7 cells for 24 and 48 hours. The cells were collected and analyzed for expression of CD44^+^ CD24^low^ and tested for mammosphere formation. At both time points the CD44^+^ CD24^low^ expressing population and number of mammosphere formed were significantly increased following exposure to the ligand.

**Table 2:** Effects of the Notch receptor inhibitor DAPT on CD44^+^ CD24^low^ expression and mammosphere formation.

DAPT Treatment (Hours)	Fold Change Mammosphere formation	Fold Change CD44^+^ CD24^low^
48	0.75±0.19*	0.37±0.13*

* p<0.05, data collected from 3 independent experiments and represented as mean ± SEM.

The gamma secretase inhibitor, DAPT, was added to culture of MCF7 cells for 48 hours. The cells were collected and analysed for expression of CD44^+^ CD24^low^ and tested for mammosphere formation. The CD44^+^ CD24^low^ expressing population and number of mammosphere formed were significantly decreased following exposure to the inhibitor.

**Figure 4:**
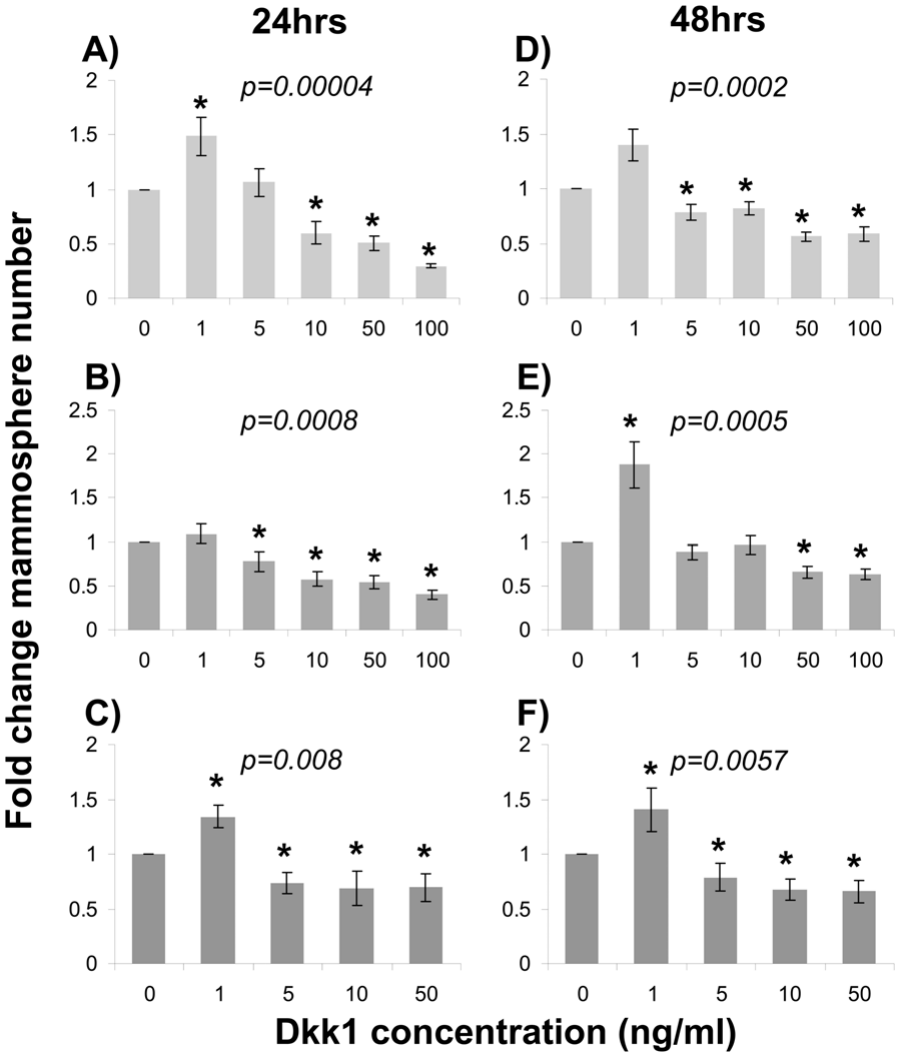
Dkk1 affects mammosphere formation by MCF-7 cells. MCF-7 cells were pre-incubated with graded concentrations of Dkk1 (0, 1, 5, 10, 50, 100 ng/mL) in serum free medium for 24 or 48 h, and then plated for mammosphere forming assay for seven days. For cells preincubated for 48 hours, Dkk1 was refreshed after 24 hours of preincubation. In the course of the seven–day incubation in mammosphere medium, Dkk1 was either absent (panels A, D), introduced at pretreatment concentration once at the beginning of the assay (panels B,E) or refreshed thereafter every 24 hours (panels C,F). *P* values for the overall variance of all data points were generated by Kruskall–Wallis test. Asterisks mark statistically significantly different (*p*≤0.05) individual comparisons of Dkk1–treated cells and Dkk1–free controls (Mann–Whitney U test).

**Figure 5:**
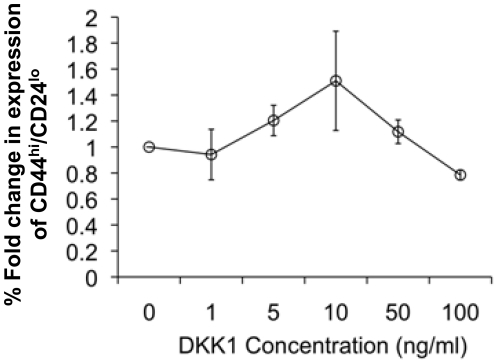
Dkk1 affects CD44^+^CD24^-/low^ expression in MCF-7 cells. MCF-7 cells were incubated with graded concentrations of Dkk1 (0, 1, 5, 10, 50, 100 ng/mL) in serum free medium for 48 h, Dkk1 was refreshed after 24 hours of incubation. Percentage of CD44^+^CD24^-/low^ cells was then analysed by flow cytometry and fold change compared to DKK-free control calculated. *P* values were generated by individual comparisons of Dkk1–treated cells and Dkk1–free controls (Mann–Whitney U test).

**Figure 6:**
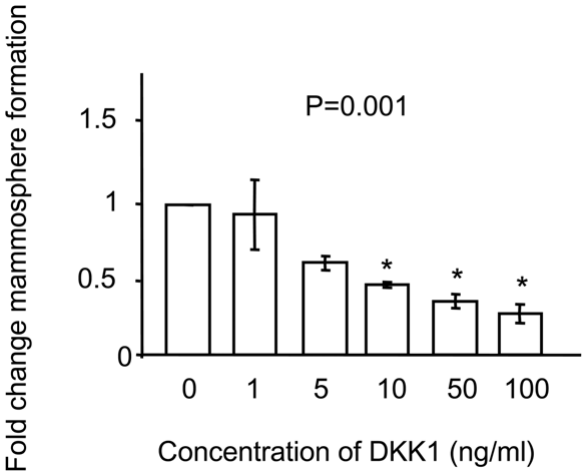
Effect of Dkk1 treatment on mammosphere formation by primary human invasive breast cancer cells. Non-adherent breast cancer cells were plated in the presence of Dkk1 (0, 1, 5, 10, 50, 100 ng/mL) and cultured for 7 days of mammosphere forming assay. Data for each concentration of Dkk1 are expressed as the fold change in mammosphere formation compared to untreated controls (0 ng/mL). P values were generated by ANOVA. Asterisks mark statistically significant differences (p≤0.05 by I-test).

When the mathematical model was used to simulate Wnt signaling effects on BC-SCs, it predicted that low Dkk1 concentrations should not affect, or somewhat increase SC proliferation, whereas above a threshold of Dkk1 concentration the BC-SC number would decrease, depending on the dose of the exogenously applied Dkk1 (Fig 2). To test this prediction, we treated MCF-7 breast cancer cells by graded doses of Dkk1 applied at schedules detailed in Materials and Methods. Under all conditions, Dkk1 stimulated MS formation at the concentration of 1.0 ng/mL while a Dkk1 concentration equal to or larger than 5.0 ng/mL consistently reduced MS formation (Fig. 4). For example, a single exposure to 1 ng/mL Dkk1 stimulated MS formation by 1.5–fold compared to untreated, while single exposure to 100 ng/mL Dkk1 reduced MS formation to 30 percent of controls (Fig. 4A). We observed this dose–response pattern under all regimens of Dkk1 introduction to cells, suggesting that multiple applications were redundant (Figs 4E–F). Whereas the biphasic effects on MS formation are significant, Dkk1 only significantly reduced CD44^+^CD24^-/low^ expression in MCF-7 cells at high concentrations (100 ng/mL; *p*<0.02, Fig. 5), whereas the increased expression at lower concentrations is more variable. Since the ER+ MCF-7 cell line was established more than 30 years ago, we wished to test our findings on fresh primary ER+ human tumor samples. We therefore tested MS formation from ER+ primary BC cells from three patient samples and demonstrated that, in contrast to the MCF-7, low Dkk1 concentrations do not affect MS formation but higher concentrations significantly reduce MS numbers (Fig. 6; *p*<0.001). This suggests that ER+ primary breast cancer cells are dependent on the Wnt pathway for SC activity as measured by the MS assay.

## Discussion

In this study we investigated how manipulation of molecular signaling pathways can influence BC-SC fate decision, potentially yielding therapeutic benefit. To this end we constructed a multi-scale mathematical model, which integrates intra-cellular and extra-cellular signal transduction in normal and malignant mammary tissues. Our preliminary analysis suggested that Dkk1 serves as anti-proliferative and pro-maturation signal in the context of a single cell [12]. Yet, it was not *a priori* evident that the same effect would be preserved when the dynamics of multiple cells at the tissue scale was to be examined. This is due to the activity of multiple, non synchronous feedbacks in the larger scale system, which could drive it to an unexpected outcome. In addition, in contrast to the relatively simple effect of Dkk1 on a single isolated SC, in tissue the effect can be non-monotonic and dose-dependent, having clear qualitative thresholds. Therefore, it was important to test what will be the influence of exogenous Dkk1 on the fate of SCs that function within the tissue environment.

Simulations of our model point to Dkk1 as a plausible mediator of SC fate decision, which can switch tissue BC-SCs from replication to differentiation in a dose-dependent manner. Our results suggest the existence of a critical Dkk1 concentration, below which SC replication remains largely unaffected or, even, slightly increases, and above which it is suppressed. A relatively small change in Wnt or Notch activity, appears to alter the Dkk1 threshold values and the Dkk1 levels that are required for SC proliferation to be reduced to zero.

These predictions were experimentally tested by checking the effects of Dkk1 on MS formation and on the proportions of cells bearing the BC-SC phenotype CD44^+^CD24^-/low^. In accordance with model predictions, low levels of Dkk1 (less than 10 ng/ml) do not significantly change the number of MS formed in ER+ BC cells from three primary samples primary BC, while doses of 10ng/ml or higher significantly reduce the number of MS formed by these cells, representing SC counts. The model is then validated by results in cells taken from primary BC cells.

Comparatively large Dkk1 concentrations reduced BC-SC numbers down to 25 percent of controls in MCF-7 cells, whereas MCF7 cells differed from primary tumor cells in response to low dose Dkk1, even though these cells were of the same ER+ breast cancer phenotype. Dosing Dkk1, 1ng/ml, significantly increased mammosphere formation in four out of six MCF7 experiments. Several explanations, relating to the assumed model parameter values, may account for this phenomenon. Possibly, such strong effect can be observed in our model under parameter values that are different from those we estimated. However, as in our study we wished to focus the investigation on the problem of fate decision, we kept other parameters of the system constant throughout the simulations and preferred not to investigate putative effects on other elements of the system, such as the differentiated cell mortality (but see, e.g[14]).

Higher Dkk1 doses were necessary for driving a similar pattern of change in the percentage of CD44^+^CD24^-/low^, but here the effect was more variable, probably due to lower sensitivity of this assay, so that only the reduction of BC-SC markers under high Dkk1 concentrations showed statistical significance. Over all, these results imply that tissue differentiation requires high doses of Dkk1. To directly demonstrate that Dkk1 at high concentrations induces mammary differentiation we plan to look at normal primary mammary cells, where markers of differentiation are well defined. We believe that this experiment will provide more conclusive information than our initial experiments.

Although the model predicts that high Dkk1 concentrations completely eliminate SCs from the cell culture, we observed no BC-SC eradication. Note, however, that according to model predictions, the dose of Dkk1, which is required for SC elimination, largely depends on the mutations leading to the changes in activity of Wnt, Notch or E-cadherin; a change of 15% in Wnt or Notch activity would almost double the Dkk1 levels required for SC proliferation to be reduced to zero. As there is no information about the genetic make-up of MCF7 cells and primary cancer samples used in the experiments, our simulations attempted to capture only the qualitative pattern of SC growth. Moreover, the difficulty to obtain pure BC-SCs and culture them without change of phenotype and function, render the evaluation of cancer SCs parameters challenging, at present. This technological problem also impinges on the present ability to retrieve the quantitative effect of Dkk1 on SC growth.

In distinction to the present system, a recently developed mathematical model of Wnt signaling derived the parameter values from measurements in the more accessible Xenopus oocytes [20]. However, since the quantitative cause–effect relationships of Dkk1 are context dependent (viz. quorum sensing), in our model construction we were limited by experimental methods applicable to BC–SCs.

Recent studies show that introduction of Dkk1 to lag–phase mesenchymal SCs, stimulates proliferation [21], and that Dkk1 allows SCs from bone marrow stroma to re-enter the cell cycle by inhibiting the Wnt/β-catenin signaling pathway [7]. In contrast to stimulating proliferation, Dkk1 appears to induce neural differentiation of embryonic SCs [22] and differentiation of colon cancer cells [23]. Our results may resolve these contradicting views of Dkk1 effect, as they suggest that the observed discrepancies reported in the literature result from experiments conducted in different regions of Dkk1 concentration–effect curves. Our model predictions are further corroborated by recent experiments suggesting that the expression levels of Dkk1 were down-regulated in LM-MCF-7 cells, as compared to MCF7 cells, and that as a result, LM-MCF-7 cells have relatively high proliferation ability, via losing control of β-catenin/TCF transcription cascade. It has been argued that over-expression of Dkk1 may be used in gene therapy to inhibit the development of BC cells [24].

Our model assumes that the Notch and the Wnt signaling pathways are linked through the inhibition of the Notch receptor degradation by activated LEF/TCF. This assumption is yet to be experimentally supported for BC cells. Implementing it in our mathematical model is justified by indirect evidence (see Materials and Methods), and backed by our conviction that these two pathways cannot be independent, as is customarily believed, but, rather, that a link between the two pathways must exist, since the transition between proliferation and differentiation should be balanced.

Another model prediction is that increasing the rate of Notch receptor synthesis increases SC proliferation in a dose dependent manner (see SI for precise assumption and formulation). This is seen in our simulations, and is explained by the model's assumption that cell's fate decision is made according to the relation between the amounts of PF and DF: the higher Notch activity gets, the lower is the rate of increase in the amounts of DF relative to the rate of increase in the amounts of PF. As a result, PF within SCs will tend to reach the proliferation threshold increasingly quicker than DF, and population proliferation rate will increase.

To verify this model prediction we mimicked an increase in Notch receptor activity by addition of rhDLL4. This resulted in augmented MCF-7 cell line MS formation and an increase in the numbers of CD44+CD24-/low cells. We tested the power of these results by two further experiments: first, we studied the effect of DAPT, which blocks Notch receptor activity, on BC-SC proliferation and showed that it was significantly reduced. Secondly, we knocked down Notch4 expression by siRNA and showed that the CD44+CD24-/low population and number of MSs formed were significantly reduced. Similar experimental results have been reported by us previously [19], but here we provide a model predicting these phenomena, an underlying mechanism explaining them, and an experimental support for their existence. We are aware that adding increasing concentrations of the Notch ligand to mimic the dys-regulation found in cancer cells may seem somewhat awkward, since it is unclear that the addition of exogenous ligands simulates the molecular defects found in primary breast cancers. However, the reported results of the DAPT and the siRNA experiments provide a support for the model's prediction about the relevance of Notch signaling to proliferation of cancer cells. Collectively, these results establish that the number of CSCs increases or decreases in accordance with the activation or inhibition of the Notch pathway, respectively, as predicted by the mathematical model and in agreement with our previous studies.

Mutations and gene methylation changes leading to aberrant Notch, Wnt and E-cadherin signaling have been found in many cancers and particularly in BC [25], [26], [27]. Here we modeled the molecular defects that increase activation of Wnt or Notch pathways or impede E-cadherin-mediated inhibition of LEF/TCF activity. Our simulations indicate that the more pronounced these defects are - particularly defects in the Notch pathway - the greater is the increase in BC-SC proliferation, and the higher is the concentration of Dkk1 that is required for SC elimination. Our model predicts that mutations that increase Notch activation by as little as 5 percent allow BC-SC proliferation at Dkk1 concentrations that induce differentiation of normal SCs (Fig. 2). This implies a mechanism by which BC-SCs, the key component of minimal residual disease, can replace the nonmalignant SCs in their niche, *e.g.*, bones [28], [29], [30].

To sum up, our mathematical model is a new multi-scale model for describing SC fate decision in developing tissues, which combines intracellular pathways with replication/differentiation behavior within the cell population. The model provides a general theory for the role of Dkk1 in SC proliferation, suggesting that Dkk1 may be the molecular mechanism mediating SC fate decision and that this is done by a QS mechanism. Specifically, the model predicts that Dkk1 controls the balance between proliferation and differentiation in individual cells, rather than inhibiting proliferation alone, and that the addition of small doses of exogenous Dkk1 does not decrease, and sometimes even increase, mammary cell proliferation, whereas large doses of Dkk1 will divert proliferating SCs to differentiation. Furthermore, the model predicts that increasing the rate of Notch synthesis increases SC proliferation, *per se,* rather than just reducing differentiation. We present herein laboratory experiments in MCF-7 cells and in ER+ BC cells biopsied from patients, which prospectively verify the mathematical model.

Our results suggest that large Dkk1 boluses applied *in situ* may serve for differentiation therapy. Such differentiation therapy has been suggested by Sell and others [31]. Here we present the theoretical basis for this therapy modality. Our study implies that Dkk1 therapy may be more robust than other methods, as it challenges a general cellular homeostasis mechanism, namely, fate decision by QS. Inappropriate Dkk1 administration may be counterproductive, as it may stimulate CSC proliferation. These *in vitro* findings will have to be verified by limiting dilution transplantation experiments on BC-SC activity in mouse xenograft models. Hopefully, this work will provide an impetus for careful examination of differentiation–directed strategies for BC therapy.

## Materials and Methods

### Model simulations

To describe a reasonably sized tissue we used the honeycomb grid on closed surface (determining that each cell may have up to 6 adjacent neighbors) with 800 cells (the dimensions of the grid were 40×20 cells). This architecture does not allow the edges to affect the system's behavior; it minimizes the effect of grid's size and has been found sufficient for exploring the properties of quorum sensing. It should be noted again here that our main concern in this work was to study what may be the mechanism of fate decision and not to analyze the specific cellular automata structure. This is done elsewhere (in preparation).

The tissue model grid was initially seeded by 36 randomly placed SCs and was simulated over three months immediately following Dkk1 treatment. The average number of SCs, over the last three simulated days, was calculated over 50 simulation experiments for each combination of concentration and cell type. It should be emphasized that the model does not make any specific assumption on the spatial distribution of SCs; the system's evolution is directed only by its stated underlying developmental rules, so that the final number of SCs in the system is independent of the specific pattern of SC position. Moreover, because the focus of this model is on the combined intracellular/extracellular effects on SC's fate decision, the independent properties of the tissue model are not explore here; for their investigation see [14] and in preparation).

The mathematical model was numerically simulated by calculation of per-cell expression levels of all modeled proteins for the duration of the simulated process and calculation of the intra-cellular dynamics for all the cells.

### Calculation of model parameters

Initial parameters for normal mammary SCs were chosen such that tissue survival to confluence is guaranteed (see Table S1, listing all the parameter values of the model).

No experimental data were found in the literature for relating the quantitative effects of mutations to changes in expression of the signal transduction proteins in breast cancer cells. Yet, since our aim was to get a qualiitative understanding of such effects, we chose representative values for each considered mutation of the “wild type,” which lead to 2-4-fold increase in SC numbers, as a rough approximation to putative oncogenic mutations. For this reason, defects in signaling pathways were represented by an increase of 5 to 20 percent in Notch receptor synthesis, or in Wnt ligand expression or in E-cadherin concentration required for inhibition of LEF/TCF activity.

To match up the model–specific units of Dkk1 to the exogenously applied Dkk1 in our experiments, we relied on an experiment in which 10^5^ MCF-7 cells were seeded in volume of 200 µl for 24 hours. The medium contained 38432 pg/ml Dkk, measured by ELISA [32]. To calculate the total volume of the medium into which Dkk1 was secreted in our simulations the representative medium volume of 2·10^−3^ µl/cell was multiplied by the number of cells in each simulation at steady state. Using average results from 50 simulations, we derived the model–specific Dkk1 unit as 2.01 ng/mL.

### Cell lines

MCF-7 cells (American Type Culture Collection, Manassas, VA) were maintained in DMEM with 10% fetal calf serum, 100 U/mL penicillin, 100 µg/mL streptomycin, and 29.2 mg/mL L-glutamine (all from Gibco, Carlsbad, CA). Cells were passaged when reached 90 percent confluency by trypsinization and reseeding at 20 percent of initial density.

### Primary invasive human BC cells

Pleural effusion samples were collected with informed consent (COREC #05/Q1402/25) from 3 patients suffering from estrogen receptor-positive (ER+) metastatic BC. Cells were collected by centrifugation at 800 ×g for 5 minutes and resuspended in PBS. Blood cells were removed by centrifugation of the cell suspension through Lymphoprep solution (Axis Shield; Norton, MA) at 800 ×g for 20 minutes. Then the remaining leukocytes cells were removed by CD45–specific immunomagnetic adsorption; first, the cells were resuspended at ≤1×10^6^ in 100 µL PBS containing 0.5 percent BSA, 2.0 mM EDTA (sorting buffer) and incubated with immunomagnetic reagent specific for human CD45 (1∶10, Miltenyi Biotec, Auburn, CA) for 15 minutes at 40°C. Cells were then washed and resuspended in 500 µL of sorting buffer, passed through the magnetic column of an AutoMACS apparatus running under the normal of DEPLETES program and the CD45–negative cells were collected. Finally, the cells were incubated with 100 µL of Dead Cell Removal microbeads and the dead cells were removed using an MS column on a MACS seperator (all from Miltenyi Biotec). Live cells were collected, resuspended in the MS medium (DMEM:F12) supplemented with B27 and MEGM SingleQuots (human epidermal growth factor, insulin, hydrocortisone, and GA-1000; Gibco). All procedures involving human samples were approved by the Local Research Ethics Committee.

### Mammosphere forming assay

A single cell suspension was prepared from MCF-7 monolayer culture using enzymatic digestion (Trypsin–EDTA, Gibco) and mechanical disaggregation by repeated passing through a gauge 25 needle. The cells were plated at a density of 500 cells/cm^2^ in culture flasks coated with 2-hydroxyethylmethacrylate; poly-HEMA (Sigma) to prevent cell anchorage. Cells were grown for seven days in the MS medium in a humidified atmosphere of 5% CO_2_ at 37°C. Spheres over 50 µM were counted. Assuming that each MS was the clonal product of a single BC–SC, we divided the number of MSs with the total numbers of cells plated and derived the MS forming cell fraction (MF). Primary breast cancer cell MS were prepared and counted in a similar way.

### Flow cytometry of CD44^+^CD24^-/low^ cells

Trypsinized MCF-7 cells were resuspended at ≤1×10^6^ cells/100 µL PBS containing 2.0 mM EDTA and 0.5 percent BSA (“buffer”) and incubated with fluorescein–conjugated antibodies specific for CD44 and phycoerythrin–conjugated antibody specific for CD24 (both at 1∶10 dilution, Beckman Coulter, High Wycombe, UK) at 40°C for 10 min. Following incubation, the cells were washed in PBS and centrifuged at 800×g for two minutes. Cells were resuspended in 500 µL of the buffer and fluorescence was measured using a FACSCalibur flow cytometer (Becton Dickinson, Oxford, UK). The data were analyzed with the aid of WinMDI 2.8 software (Scripps Institute, San Diego, CA).

### NOTCH pathway activation and inhibition

To quantify Delta4 activation, 100 µg/mL rhDLL4 (R&D Systems, Abingdon, UK) was dissolved in PBS containing 0.1 percent BSA and stored at −20°C. One day before use, the stock solution of rhDLL4 was diluted 1∶100 in PBS containing 0.2 percent gelatine (Sigma) and used to coat T75 flasks (Gibco, Carlsbad, CA). The flasks were then incubated at 40°C overnight. The next day, the solution was removed and 3×10^6^ MCF-7 cells were plated, grown for 24 to 48 h, then taken for MS forming assay and flow cytometry of CD44^+^CD24^-/low^ cells.

To determine the effects of γ-secretase on SC proliferation, γ-secretase inhibitor DAPT (N-[N-(3,5-difluorophenacetyl-l-alanyl)]-S-phenylglycine t-butyl ester; Calbiochem, Nottingham, UK) was added at 10 µM to adherent cells or MSs at the day of plating. Controls were treated with 0.001 percent DMSO (Sigma).

siRNAs designed to interfere with four unique sites in the Notch4 receptor were used from the ON_TARGETplus set (Dharmacon, Lafayette CO; siRNA ID and sequence: J-011883-05–GCACGGACGGUGUCAGUAA, J-011883-06–GCAGGAGGGUCCA CGUUGU, J-011883-07–GGUGAGACGUGCCAGUUUC, J-011883-08–GCCCAACCCUGC GAUAAU-G). One million MCF-7 cells were plated 24 h before transfection with siRNA primers. Briefly, sequence-specific siRNA primers and a scrambled sequence Control were diluted to 20 µM. Dharmafect4 transfection reagent was made up in Optimem serum–free medium and added to cells for 72 h as recommended by the manufacturer.

### Wnt pathway - Dkk1 effect

One million MCF-7 cells were cultured in T75 flasks for 48 h when they were washed with PBS. Then the cells were incubated in serum–free medium [phenol-red–free DMEM (Gibco) with penicillin/streptomycin and L-glutamine] for 24 h. Cells were further treated with Dkk1 at 1, 5, 10, 50 or 100 ng/mL for 24 h or 48 h and trypsinized (0.125 percent Trypsin-EDTA; Worthington Biochemical Corporation, Lakewood, NJ) immediately thereafter. To maintain Dkk1 concentration in culture, the medium was refreshed daily. Collected cells were taken for MS forming assay and flow cytometry of CD44^+^CD24^-/low^ cells. In the MS forming assay cells were plated in the MS medium for seven days (i) in the absence of Dkk, (ii) with a single addition of Dkk1 or (iii) with medium refreshment Dkk1 every 24 h.

### Statistical Analysis

Normally distributed data were analyzed by the analysis of variance to determine significant differences of Dkk1 treatment followed by individual comparisons to Control (no Dkk1) using a two-sided t-test. Data not distributed normally were analyzed by the Kruskall-Wallis test followed by individual comparisons to normal using the Mann–Whitney U test. *P* values equal to and less than 0.05 were taken to indicate statistically significant differences.

## Supporting Information

Text S1 **The assumptions underlying all the model equations and their biological justification, as well as all the model equations and their derivation.**
(DOC)

Table S1 **The parameter values used in tmodel simulations.** For parameter definitions see text.
(DOC)
