# Supplementary material for: Dickkopf1 Regulates Fate Decision and Drives Breast Cancer Stem Cells to Differentiation: An Experimentally Supported Mathematical Model
Source: PLoS One. 2011 Sep 6;6(9):e24225. doi: 10.1371/journal.pone.0024225 (PMC3167819; doi:10.1371/journal.pone.0024225)
Supplement: Text S1 — The assumptions underlying all the model equations and their biological justification, as well as all the model equations and their derivation. (DOC) [file pone.0024225.s001.doc]

**Supporting Information Text S1**

**Biological background for the mathematical model.** Self-renewal of mammary SCs is mediated by different signaling pathways, including Wnt [1,2,3], Notch [4] and E-cadherin [5]. The Wnt pathway is activated by binding the Wnt ligands to Frizzled/LRP membrane receptors, leading to nuclear accumulation of β-catenin [6,7], which, in association with the transcription factors LEF/TCF, induces transcription of target genes, and brings about cell proliferation [8]. Absence of nuclear β-catenin may lead to cellular differentiation [9,10]. Dkk 1 is a target gene of the LEF/TCF transcription factor [11].

Notch signaling is also involved in the mammary SC self-renewal [4]. A sequence of molecular events that follow the binding of the transmembrane ligands, Delta, Serrate, Lag-2 (DSL), lead to up-regulation of the expression of genes, such as Hes, which suppress differentiation [12]. Notch signaling pathway can be inhibited by the binding of Numb to its intracellular domain [13].

It is likely that Notch and Wnt signaling pathways are not independent. Their interaction can plausibly be mediated, for example, through the promoter region of Numb, which comprises two binding sites for LEF/TCF [14], or through the Notch ligand, Jag1, known to be upregulated by activation of LEF/TCF [15]. Another possible Wnt/Notch mediator is the Musashi1 protein, induced by Wnt. This protein increases the efficiency of Notch signaling by transcriptional inhibition of the Notch inhibitor Numb [16].

The aforementioned translocation of β-catenin is also involved also in regulation of the adhesion molecule E-cadherin, which mediates SC contacts with neighboring cells [17]. β-catenin mediates the association of E-cadherin with the cytoskeleton, affecting the efficiency of transcription induced by the β-catenin/LEF/TCF complex [18]. Expression of E-cadherins is enhanced during cell differentiation, whereas its repression is associated with epithelial-mesenchymal transition, more abundant cancer SC–like cells and carcinogenesis [5]. Association of defective Wnt and Notch signaling with BC points to their possible role in carcinogenesis [19].

Numerous abnormalities in the Wnt pathway have been associated with increased expression of upstream components of Wnt ligands, Fzd receptors, or with epigenetic inactivation of secreted negative regulators [20]. Similarly, high levels of Notch ligands DLL1, JAG1 and JAG2 and Notch receptor genes were expressed in BC and were aberrantly activated [21,22]. Notably, aberrantly high levels of Notch ligand and receptor correlated with poor overall survival of BC patients [22].

**Mathematical model of breast cancer stem cells in a cancerous tissue**

**Stem Cell (SC) model**

In our model, the influence of a protein on the state of another protein is described using a non-negative Hill functions of the form

$$f\left(x\right)=\frac{u\cdot a^{m}+v\cdot x^{m}}{a^{m}+x^{m}},$$

where $u=f\left(0\right),$ $v=\lim_{x\to+\infty} f\left(x\right),$ $a=f^{-1}\left(\frac{u+v}{2}\right),$ and $m$ is the steepness of the function. These functions are of sigmoid shape, herein referred to as *sigmoids*. Different sigmoids will be denoted by subscripts ($f_{F}$, $f_{S}$, etc.). The parameters, used in these sigmoids will have the corresponded subscripts. We also denote an increasing function by $f^{\uparrow}$, and a decreasing fuction by $f^{\downarrow}$.

**Wnt pathway**

We assume that Wnt signal intensity depends on the total expression levels of Wnt and Dkk1 proteins in the closest environment. The Wnt protein activates the Wnt pathway whereas the Dkk1 protein forms a negative feedback loop in the Wnt pathway. The Wnt signal intensity is determined by the following equation

$$S\left(t\right)=f_{F}^{\uparrow}\left(W_{t}\left(t\right)\right)\cdot f_{S}^{\downarrow}\left(D_{t}\left(t\right)\right),$$

where

- $S$ is the Wnt signal intensity;
- $W_{t}$ and $D_{t}$ are the total expression levels of Wnt and Dkk1 proteins, respectively, in the adjacent environment of the considered cell;
- $f_{F}$ represents Wnt signal intensity as a function of $W_{t}$ in the absence of Dkk1;
- $f_{S}$ represents a coefficient of Wnt signaling inhibition by Dkk1, as a function of $D_{t}$ (0 stands for absolute inhibition of Wnt signaling, while 1 stands for absolute non-inhibition).

Activation of the transcription factors LEF/TCF is positively controlled by the Wnt signal intensity and negatively controlled by the E-cadherins that are bound to neighboring cells. Deactivation of the transcription factors LEF/TCF is proportional to the level of activated LEF/TCF. Thus the kinetics of activated LEF/TCF is determined by the following equation:

where $L$ is the activated LEF/TCF, $E_{b}$ is the E-cadherins that are bound to neighboring cells, $f_{L}$ is the dependence of LEF/TCF activation rate on the bound E-cadherin influence, and $\mu_{L}$ is the LEF/TCF degradation rate.

The synthesis of Dkk1 is positively regulated by the activated LEF/TCF transcription factors within the cell whereas, the degradation of Dkk1 is proportional to its expression level. Thus the kinetics of the Dkk1 produced by particular cell is determined by the following equation:

where $D$ is the Dkk1 produced by the particular cell, $f_{D}$ is the dependence of the Dkk1 synthesis rate on $L$, and $\mu_{D}$ is the Dkk1 degradation rate.

Dkk1 protein level in the closest environment of a SC is comprised of Dkk1 secreted from the mentioned SC and Dkk1 secreted from neighboring SCs. In addition, we take into consideration the geometrical structure implying the definition of neighborhood in the model. Thus, we use the following formula to calculate the Dkk1 protein level in the closest environment of a SC:

$D_{t}=\frac{1}{2}\cdot\left(D+\frac{D_{n}}{6}\right),$ (1)

where $D_{n}$ is the sum of the Dkk1 produced by all neighbouring stem cells in the closest environment of the SC. Here and in all equations below, 6 is a parameter chosen to represent the maximal number of cell's adjacent neighbors (see below).

The Wnt protein level in the closest environment of a SC is comprised of Wnt secreted from the mentioned SC and Wnt secreted from neighboring cells including the SCs and differentiated cells, DCs. Wnt secretion level is constant for all cells. The equation for Wnt protein level is similar to the equation for Dkk1 protein level which is mentioned above in the Equation (1):

$$W_{t}=\frac{1}{2}\cdot\left(W+\frac{W_{n}}{6}\right),$$

where $W$ is the Wnt secreted from the particular cell, and $W_{n}$ is the sum of all levels of Wnt secreted from all neighbouring cells in the closest environment.

In the present model cell proliferation is induced by the proliferation factors (PFs). The synthesis of PFs is up-regulated by the activated LEF/TCF transcription factors. Degradation of PFs is proportional to their expression level (first order degradation). Thus, the kinetics of PFs is satisfies the equation:

where $P$ is the level of PFs, $f_{P}$ is the dependence of the PF synthesis rate on $L$, and $\mu_{P}$ is the PF degradation rate.

**E-cadherin**

Our model assumes that E-cadherin synthesis is negatively regulated by Wnt signal intensity. E-cadherin degradation is proportional to their expression level. Hence, the kinetics of E-cadherins before binding to neighboring cells is determined by the equation:

where $E$ is the E-cadherin level, $f_{E}$ is the dependence of E-cadherin synthesis rate on $S$, and $\mu_{E}$ is the E-cadherin degradation rate.

Of note: we use the equation for describing the kinetics of E-cadherins in DC by substituting value of $S$ with 0, resulting in increased expression of E-cadherins in DC.

The description of the binding of E-cadherins to an adjacent cell is based on the following assumptions:

1. binding of E-cadherins to adjacent cell is proportional to E-cadherins levels of both the considered cell and that adjacent cell.
2. the level of E-cadherins bound to adjacent cell does not decrease unless one of these two cells dies.

The kinetics of E-cadherins that are bound to the adjacent cell are determined by the following equation:

$$E_{b,i}(t)=\max\left(E_{b,i}\left(0\right),\max_{\tau\leq t} \left(k_{b}\cdot E\left(\tau\right)\cdot E_{i}\left(\tau\right)\right)\right),$$

where $E_{b,i}$ is the level of the bound E-cadherins to the neighbouring cell into $i$-th direction, $E_{i}$ is the level of E-cadherins in the neighbouring cell being in $i$-th direction from the considered cell, and $k_{b}$ is the E-cadherin binding coefficient.

The total level of bound E-cadherins is the sum of bound E-cadherins to the all adjacent cells:

$$E_{b}\left(t\right)=\sum_{i=1}^{6} E_{b,i}\left(t\right).$$

**Notch pathway**

In the current model the Notch pathway is active in SCs. The proteins involved in this pathway are zeroed at the moment a SC is differentiated. Notch receptor synthesis is constant (zero order synthesis). Notch receptor degradation is inhibited by activated LEF/TCF. Thus, the kinetics of Notch receptor is determined by the following equation:

where $N$ is the Notch receptor level, $p_{N}$ is the Notch receptor synthesis rate, and $f_{N}$ is the dependence of Notch receptor degradation rate on $L$.

Every cell in the model expresses the DSL proteins, Notch ligands, in a constant level. The amount of Notch receptors in a particular SC, which are ready to be activated, is the minimum between two values: the available Notch receptor in the SC and the total DSL directed to it (1/6 from the total expression of DSL in adjacent cells to the SC). The amount of Notch receptors ready to be activated is calculated as the following:

$$\begin{matrix} N_{l}=\frac{1}{6}\cdot\sum_{i=1}^{6} N_{l,i}, \\ N_{r}=\min\left(N,N_{l}\right), \end{matrix}$$

where $N_{l}$ is the total level of DSL directed to the cell, $N_{l,i}$ is the DSL level of the neighbouring cell being in $i$-th direction from the considered cell, and $N_{r}$ is the level of Notch receptor ready to be activated.

Hes protein synthesis is positively regulated by the activated Notch receptor. Hes degradation is proportional to its expression level. Thus the kinetics of Hes proteins is determined by the following equation:

where $H$ is the HES level, $f_{H}$ is the dependence of the HES synthesis rate on $N_{r}$, and $\mu_{H}$ is the HES degradation rate.

In the present model cell differentiation is induced by the differentiation factors (DFs). Up-regulation of DFs synthesis is negatively regulated by Hes expression. Degradation of DFs is proportional to their expression level. Thus, the kinetics of DFs is satisfies the equation:

where $M$ is the level of DFs, $f_{M}$ is the dependence of the DF synthesis rate on $H$, and $\mu_{M}$ is the DF degradation rate.

The present model takes into consideration the following assumptions regarding cell fate.

1. When DFs reach to a certain threshold $c_{M}$ the SC will differentiate.
2. When PFs are greater than or equal to a certain threshold $c_{P}$ and there is a non-occupied site adjacent to it then the cell will proliferate following a certain time mentioned above. At the time the cell waits to proliferate, the values of DFs and PFs are nullified.

**References**

1. Brennan KR, Brown AM (2004) Wnt proteins in mammary development and cancer. J Mammary Gland Biol Neoplasia 9: 119-131.

2. Dontu G, Jackson KW, McNicholas E, Kawamura MJ, Abdallah WM, et al. (2004) Role of Notch signaling in cell-fate determination of human mammary stem/progenitor cells. Breast Cancer Res 6: R605-615.

3. Reya T, Clevers H (2005) Wnt signalling in stem cells and cancer. Nature 434: 843-850.

4. Harrison H, Farnie G, Howell SJ, Rock RE, Stylianou S, et al. (2010) Regulation of Breast Cancer Stem Cell Activity by Signalling Through the Notch4 Receptor. Cancer Res.

5. Gupta PB, Onder TT, Jiang G, Tao K, Kuperwasser C, et al. (2009) Identification of selective inhibitors of cancer stem cells by high-throughput screening. Cell 138: 645-659.

6. Polakis P (2000) Wnt signaling and cancer. Genes Dev 14: 1837-1851.

7. Rubinfeld B, Albert I, Porfiri E, Fiol C, Munemitsu S, et al. (1996) Binding of GSK3beta to the APC-beta-catenin complex and regulation of complex assembly. Science 272: 1023-1026.

8. Behrens J, von Kries JP, Kuhl M, Bruhn L, Wedlich D, et al. (1996) Functional interaction of beta-catenin with the transcription factor LEF-1. Nature 382: 638-642.

9. Cavallo RA, Cox RT, Moline MM, Roose J, Polevoy GA, et al. (1998) Drosophila Tcf and Groucho interact to repress Wingless signalling activity. Nature 395: 604-608.

10. Chen G, Fernandez J, Mische S, Courey AJ (1999) A functional interaction between the histone deacetylase Rpd3 and the corepressor groucho in Drosophila development. Genes Dev 13: 2218-2230.

11. Chamorro MN, Schwartz DR, Vonica A, Brivanlou AH, Cho KR, et al. (2005) FGF-20 and DKK1 are transcriptional targets of beta-catenin and FGF-20 is implicated in cancer and development. EMBO J 24: 73-84.

12. Wu F, Stutzman A, Mo YY (2007) Notch signaling and its role in breast cancer. Front Biosci 12: 4370-4383.

13. Berdnik D, Torok T, Gonzalez-Gaitan M, Knoblich JA (2002) The endocytic protein alpha-Adaptin is required for numb-mediated asymmetric cell division in Drosophila. Dev Cell 3: 221-231.

14. Katoh M, Katoh M (2006) NUMB is a break of WNT-Notch signaling cycle. Int J Mol Med 18: 517-521.

15. Katoh M, Katoh M (2006) Notch ligand, JAG1, is evolutionarily conserved target of canonical WNT signaling pathway in progenitor cells. Int J Mol Med 17: 681-685.

16. Okano H, Kawahara H, Toriya M, Nakao K, Shibata S, et al. (2005) Function of RNA-binding protein Musashi-1 in stem cells. Exp Cell Res 306: 349-356.

17. Chen H, Paradies NE, Fedor-Chaiken M, Brackenbury R (1997) E-cadherin mediates adhesion and suppresses cell motility via distinct mechanisms. J Cell Sci 110 ( Pt 3): 345-356.

18. Huber AH, Weis WI (2001) The structure of the beta-catenin/E-cadherin complex and the molecular basis of diverse ligand recognition by beta-catenin. Cell 105: 391-402.

19. Zardawi SJ, O'Toole SA, Sutherland RL, Musgrove EA (2009) Dysregulation of Hedgehog, Wnt and Notch signalling pathways in breast cancer. Histol Histopathol 24: 385-398.

20. Barker N, Clevers H (2006) Mining the Wnt pathway for cancer therapeutics. Nat Rev Drug Discov 5: 997-1014.

21. Farnie G, Clarke RB, Spence K, Pinnock N, Brennan K, et al. (2007) Novel cell culture technique for primary ductal carcinoma in situ: role of Notch and epidermal growth factor receptor signaling pathways. J Natl Cancer Inst 99: 616-627.

22. Reedijk M, Odorcic S, Chang L, Zhang H, Miller N, et al. (2005) High-level coexpression of JAG1 and NOTCH1 is observed in human breast cancer and is associated with poor overall survival. Cancer Res 65: 8530-8537.
