# Supplementary material for: Dickkopf1 Regulates Fate Decision and Drives Breast Cancer Stem Cells to Differentiation: An Experimentally Supported Mathematical Model
Source: PLoS One. 2011 Sep 6;6(9):e24225. doi: 10.1371/journal.pone.0024225 (PMC3167819; doi:10.1371/journal.pone.0024225)
Supplement: Table S1 — The parameter values used in tmodel simulations. For parameter definitions see text. (DOC) [file pone.0024225.s002.doc]

Supporting Information Table S1

Parameter values used in the simulations. For parameter definition see text.

| Parameter | Value | Parameter | Value | Parameter | Value | Parameter | Value |
| --- | --- | --- | --- | --- | --- | --- | --- |
| Φ | 200 *h* | *a_L_* | 50 | *μ_L_* | 0.3 *h^-1^* | *u_P_* | 0 |
| τ | 12 *h* | *m_L_* | 5 | *u_F_* | 0 | *v_P_* | 30 *h^-1^* |
| *W* | 60 | *u_E_* | 20 *h^-1^* | *v_F_* | 36 | *a_P_* | 40 |
| *N_l,i_* | 90 | *v_E_* | 0 | *a_F_* | 30 | *m_P_* | 5 |
| *μ_D_* | 0.3 *h^-1^* | *a_E_* | 20 | *m_F_* | 1 | *μ_M_* | 0.3 *h^-1^* |
| *u_D_* | 0 | *m_E_* | 2 | *u_S_* | 1 | *u_M_* | 34 *h^-1^* |
| *v_D_* | 18 *h^-1^* | *μ_E_* | 0.1 *h^-1^* | *v_S_* | 0 | *v_M_* | 4 *h^-1^* |
| *a_D_* | 28 | *k_b_* | 0.0003 | *a_S_* | 35 | *a_M_* | 40 |
| *m_D_* | 1 | *μ_H_* | 0.3 *h^-1^* | *m_S_* | 2 | *m_M_* | 5 |
| *u_N_* | 0.3 *h^-1^* | *u_H_* | 0 | *u_L_* | 0.6 *h^-1^* | *C_P_* | 15 |
| *v_N_* | 0 | *v_H_* | 30 *h^-1^* | *v_L_* | 0 | *C_M_* | 30 |
| *a_N_* | 40 | *a_H_* | 40 | *p_N_* | 10 *h^-1^* | *μ_P_* | 0.25 *h^-1^* |
| *m_N_* | 5 | *m_H_* | 3 |  |  |  |  |
